# Efficacy and safety of momelotinib in Janus kinase inhibitor-experienced Asian patients with myelofibrosis and anemia

**DOI:** 10.1007/s12185-025-04037-6

**Published:** 2025-07-21

**Authors:** Sung-Soo Yoon, Chih Cheng Chen, Sung-Eun Lee, Hung Chang, June-Won Cheong, Hsin-An Hou, Won Sik Lee, Sung-Nam Lim, Joon Ho Moon, Kiat Hoe Ong, Yi Dai, Chang Liu, Jun Kawashima, Yeow Tee Goh

**Affiliations:** 1 Division of Hematology/Medical Oncology, Department of Internal Medicine, Seoul National University Hospital, Seoul National University College of Medicine, Seoul, Korea; 2 Department of Internal Medicine - Division of Hematology and Oncology Chang Gung Medical Foundation, Chiayi Chang Gung Memorial Hospital, Puzi City, Taiwan; 3 Department of Hematology, Seoul St. Mary’s Hospital, College of Medicine, The Catholic University of Korea, Seoul, Korea; 4 Department of Internal Medicine - Division of Hematology, Chang Gung Medical Foundation - Linkou Chang Gung Memorial Hospital, Taoyuan City, Taiwan; 5 Department of Medicine - Division of Hematology, Severance Hospital, Seoul, Korea; 6 Divisions of Hematology and General Medicine, Department of Internal Medicine, National Taiwan University Hospital, Taipei, Taiwan; 7 Department of Internal Medicine, Inje University Busan Paik Hospital (Cancer Center), Busan, Korea; 8 Department of Internal Medicine, Inje University College of Medicine, Haeundae Paik Hospital, Busan, Korea; 9 Department of Hematology-Oncology, Kyungpook National University Hospital (KNUH), Daegu, Korea; 10 Haematology Service, National Healthcare Group (NHG) - Tan Tock Seng Hospital (TTSH), Singapore, Singapore; 11 GSK Plc, Shanghai, China; 12 Sierra Oncology, a GSK Company, San Mateo, USA; 13 Department of Haematology, Singapore Health Services (SingHealth) - Singapore General Hospital (SGH), Singapore, Singapore

**Keywords:** Myelofibrosis, Momelotinib, Danazol, MOMENTUM, Asian

## Abstract

**Introduction:**

This post hoc analysis investigated the efficacy and safety of momelotinib in the Asian subpopulation of MOMENTUM (NCT04173494).

**Methods:**

Patients were randomized 2:1 to momelotinib 200 mg once daily (QD) plus danazol placebo (momelotinib group) or danazol 600 mg QD plus momelotinib placebo (danazol group) for 24 weeks (W), after which they could receive open-label momelotinib or danazol. Primary endpoint: W24 total symptom score (TSS) response rate (≥ 50% reduction from baseline). W24 key secondary endpoints: transfusion independence rate; mean TSS change from baseline; splenic response rate; rate of zero transfusions.

**Results:**

Seventeen Asian patients with myelofibrosis were included (momelotinib: *n* = 11; danazol: *n* = 6). TSS response rate at W24 was 36.4% with momelotinib and 0% with danazol. Secondary endpoints favored momelotinib and were consistent with the intention-to-treat population. Grade ≥ 3 treatment-emergent adverse events were reported in 36.4 and 66.7% of the momelotinib and danazol groups, respectively, including one grade ≥ 3 anemia in the momelotinib group. Treatment interruption and/or dose reduction occurred in 18.2 and 16.7% of the momelotinib and danazol groups, respectively. Two danazol-treated patients discontinued study treatment.

**Conclusion:**

In the Asian subpopulation of MOMENTUM, momelotinib improved myelofibrosis-associated symptoms, anemia measures, and spleen response, with generally favorable safety versus danazol.

**Supplementary Information:**

The online version contains supplementary material available at 10.1007/s12185-025-04037-6.

## Introduction

Myelofibrosis (MF) is a chronic, progressive myeloproliferative neoplasm characterized by bone marrow fibrosis, extramedullary hematopoiesis, and increased production of inflammatory cytokines [1–3]. Clinical manifestations include anemia, fatigue, night sweats, fever, cachexia, bone pain, pruritus, weight loss, abdominal distension, and pain associated with splenomegaly, leading to limited social and physical activity and markedly reduced quality-of-life (QoL) in patients with MF [2, 3].

Patients with MF tend to have poor prognoses. Anemia is a major risk factor for survival according to prognostic models [4, 5], with the refined Dynamic International Prognostic Scoring System (DIPSS) plus prognostic model considering anemia (hemoglobin < 10 g/dL) and transfusion dependency as independent prognostic factors [5]. At diagnosis, approximately 40% of patients have anemia, and most develop anemia with disease progression [6]. Other risk factors include acute myeloid leukemia, which occurs in 20% of individuals [2, 3], infection, hemorrhage, progressive bone marrow failure, and cardiovascular events [3, 5, 7].

MF is rare across all ethnicities. The estimated annual incidence of MF varies from 0.4 to 3.0 per 100,000 population in North America and Europe [8–11], compared with 0.15–0.9 per 100,000 population in Korea [12, 13] and 0.43 per 100,000 among the Chinese population in Singapore [14].

Constitutive activation of the Janus kinase (JAK)-signal transducers and activators of transcription (STAT) signaling pathway, which regulates the cell cycle, cytokines, and erythropoiesis, is believed to play a key role in MF pathogenesis [15, 16]. As such, JAK inhibitors have been developed for the treatment of MF and approved by regulatory agencies based on clinical benefits. While approved JAK inhibitors have shown benefits on spleen volume and symptoms, some JAK inhibitors, such as ruxolitinib, may cause or worsen anemia in patients with MF, including in Asian patients [2, 17, 18]. The management of MF-associated anemia may involve red blood cell (RBC) transfusions, prednisolone, and anabolic hormones, such as danazol, as supportive care [17].

Momelotinib, an oral inhibitor of JAK1/2 and activin A receptor type 1 (ACVR1) inhibits the ACVR1 signaling pathway in addition to the JAK–STAT pathway. As such, momelotinib can improve anemia by decreasing hepatic hepcidin expression and increasing the efficiency of iron required for erythropoiesis [19, 20]. In the phase 3 SIMPLIFY-1 and SIMPLIFY-2 trials, momelotinib was shown to reduce spleen size and symptoms, lessen anemia, and reduce transfusion dependency [21, 22]. In SIMPLIFY-1, JAK inhibitor-naïve patients were treated with momelotinib compared with ruxolitinib and met its primary endpoint of non-inferiority in reducing spleen volume by ≥ 35% at Week 24 from baseline. In SIMPLIFY-2, momelotinib was compared with best available therapy, mostly ruxolitinib, in JAK inhibitor-experienced patients and did not achieve superiority in reducing spleen volume by ≥ 35% potentially due to the lack of a post-ruxolitinib washout period [20–22]. A third redesigned phase 3 trial, MOMENTUM, was developed to fully understand the clinical profile of momelotinib in patients with MF [20].

MOMENTUM was an international, double-blind, randomized, phase 3 study to evaluate the efficacy and safety of momelotinib compared with danazol in patients with symptomatic and anemic MF who were previously treated with JAK inhibitors (funding: Sierra Oncology, Inc., a GSK company; NCT04173494) [20]. Momelotinib demonstrated clinically significant improvements in MF-associated symptoms, anemia measures, and spleen size, along with favorable safety compared with danazol [20]. This post hoc analysis investigated the efficacy and safety of momelotinib in the Asian subpopulation of the MOMENTUM trial.

## Methods

### Study design

The MOMENTUM study design has been published previously [20]. Briefly, eligible patients were randomly assigned (2:1) to receive momelotinib 200 mg orally once daily plus danazol placebo (momelotinib group) or danazol 300 mg orally twice daily plus momelotinib placebo (danazol group) for a randomized period of up to 24 weeks, after which patients in the danazol treatment group who completed the randomized period could continue to receive open-label danazol or switch to open-label momelotinib.

### Eligibility

The full eligibility criteria have been reported previously [20]. Key inclusion criteria included: aged ≥ 18 years; confirmed diagnosis of primary MF, post-polycythemia vera or post-essential thrombocythemia (post-PV/ET) MF; prior treatment with an approved JAK inhibitor for ≥ 90 days or ≥ 28 days if therapy was complicated by ≥ 4 units of RBC transfusion in 8 weeks, or grade 3/4 adverse events of thrombocytopenia, anemia, or hematoma; Myelofibrosis Symptom Assessment Form (MFSAF) Total Symptom Score (TSS) ≥ 10 at screening; anemia (hemoglobin < 10 g/dL); platelets > 25 × 10^9^ cells/L; DIPSS high, intermediate-2, or intermediate-1 risk; palpable splenomegaly ≥ 5 cm below the left costal margin at screening or ≥ 450 cm^3^ splenomegaly volume as assessed by ultrasonography, MRI, or CT.

Key exclusion criteria included: prior treatment with momelotinib, JAK inhibitor (within 1 week prior to the first day of baseline), CYP3A4 inducers, investigational agents, danazol, splenic irradiation, or current treatment with simvastatin, atorvastatin, lovastatin, or rosuvastatin; history of prostate cancer; prostate specific antigen > 4 ng/mL; prior splenectomy; uncontrolled intercurrent illness; active or chronic bleeding; unstable angina pectoris; congestive heart failure; uncontrolled cardiac arrhythmia; progressive thrombosis; QT interval corrected using Fridericia’s Formula interval > 500 ms; history of porphyria; Child–Pugh score ≥ 10; psychiatric illness; prior or concurrent malignancy; anemia; HIV; viral hepatitis; unresolved non-hematologic toxicities from prior therapies; peripheral neuropathy; pregnant or lactating.

### Endpoints

The primary endpoint of the MOMENTUM trial was TSS response rate (≥ 50% reduction in TSS from baseline at Week 24 as assessed by MFSAF v4.0). If the primary endpoint was met, hierarchical testing of the following key secondary endpoints at Week 24 were performed: transfusion independence (TI) rate (percentage of patients with no RBC transfusions in the 12 weeks prior to completion of the 24-week randomized-treatment period and no hemoglobin < 8 g/dL), splenic response rate (SRR; percentage of patients with ≥ 25% or ≥ 35% reduction in spleen volume from baseline as measured by MRI or CT), mean change in TSS from baseline, percentage of patients who did not receive RBC or whole blood transfusion during the randomized-treatment period, TI rate in patients with transfusion dependence (TD) at baseline (≥ 4 RBC units transfused in the 8 weeks prior to the first dose of study drug), overall survival (OS; the interval from the first study drug dosing date [or randomization date for participants who did not receive treatment] to death from any cause) and leukemia-free survival (LFS; the interval from first study drug dosing date [or randomization date for participants who did not receive treatment] to any evidence of leukemic transformation and/or death from any cause).

### Safety

Adverse events (AEs) were coded using the using the Medical Dictionary for Regulatory Activities and graded according to the National Center Institute Common Terminology Criteria for AEs, including treatment-emergent AEs (TEAEs; AEs occurring or worsening on or after the first dose of study treatment, and up to 30 days after the last dose of study drug received).

### Data interpretation

Results in this sub-analysis are descriptive as this Asian subgroup was defined post hoc and was not powered for statistical comparison.

## Results

### Patient disposition

**Fig. 1:**
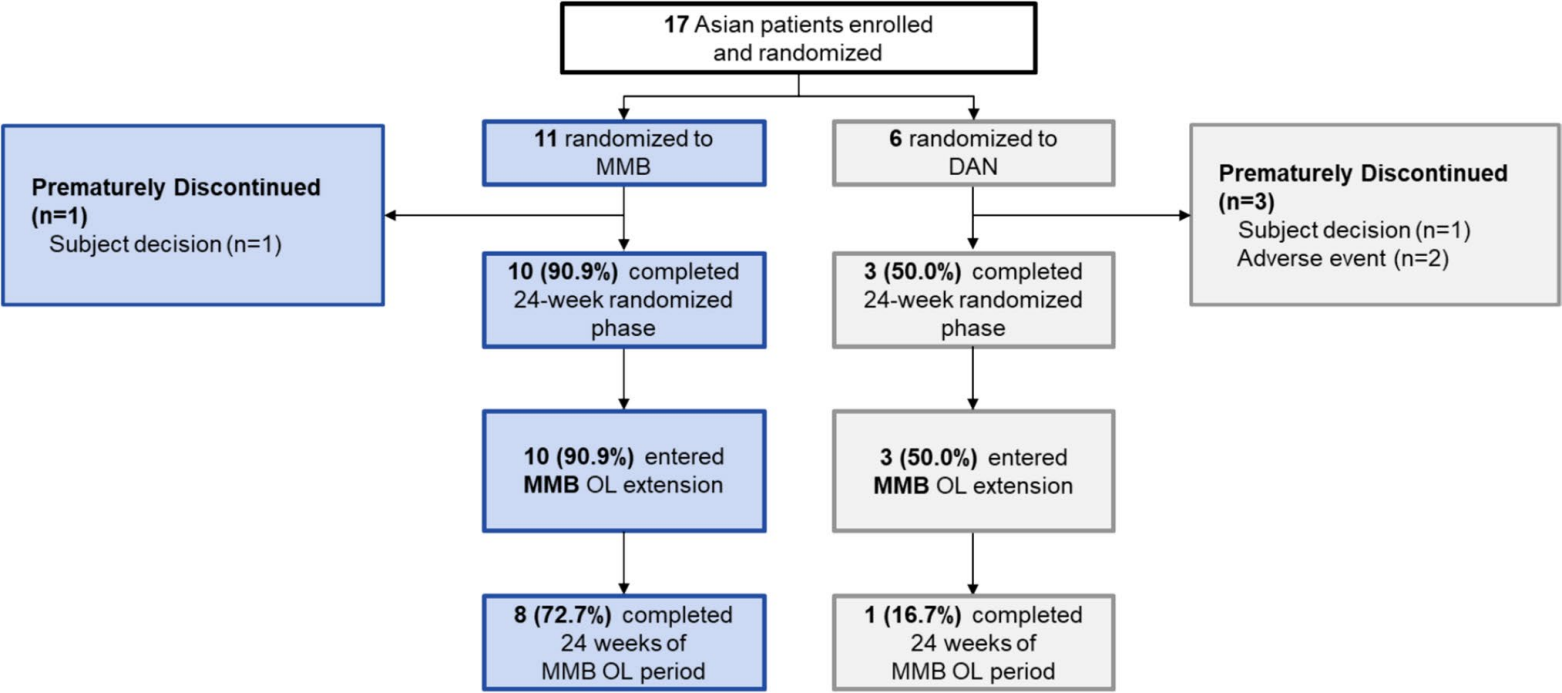
Patient disposition DAN, danazol; MMB, momelotinib; OL, open-label

From April 24, 2020, to December 3, 2021 (data cut-off date), 10 sites in Asia participated in the MOMENTUM trial [20]; 17 Asian patients with MF were enrolled (from Korea, *n* = 11 [64.7%]; Singapore, *n* = 4 [23.5%]; Taiwan, *n* = 2 [11.8%]) (Fig. 1), of whom 11 and 6 were randomly assigned to the momelotinib and danazol group, respectively, and 10 and 3 completed the 24-week randomized phase of treatment. Reasons for treatment discontinuation were AEs (0 in the momelotinib group, 2 [33.3%] in the danazol group) and subject decision (1 [9.1%] in the momelotinib group and 1 [16.7%] in the danazol group). Of the momelotinib and danazol groups, 10 and 3 patients, respectively, continued to the open-label phase and received open-label momelotinib. Eight (72.7%) patients in the momelotinib group and 1 (16.7%) patient in the danazol group completed 24 weeks of the open-label phase.

### Baseline and clinical characteristics of participants

Patient baseline and clinical characteristics are summarized in Table 1. Six (54.5%) patients were female in the momelotinib group; no patients were female in the danazol group. At baseline, 54.5% (6/11) and 50.0% (3/6) of patients in the momelotinib and danazol groups, respectively, were diagnosed with primary MF; median platelet counts were 87.0 × 10^9^/L and 89.5 × 10^9^/L, and mean hemoglobin levels were 7.9 g/dL and 7.5 g/dL. All 17 patients received prior ruxolitinib treatment for a mean (SD) duration of 134.4 (116.5) and 64.3 (67.0) weeks in the momelotinib and danazol groups, respectively; one patient in the momelotinib group also received prior fedratinib for 92.4 weeks. At baseline, 18.2% (2/11) and 9.1% (1/11) of patients in the momelotinib group were TD and TI, respectively, versus 50.0% (3/6) and 0% in the danazol group. There were small differences between the treatment groups in sex, age group, MF type, prognostic risk category, spleen volume, and RBC units transfused, but the number of patients was limited and there were no differences in other categories.

**Table 1:** Baseline demographics and clinical characteristics

		MMB (*n* = 11)	DAN (*n* = 6)
Median age at baseline, years (range)		65.00 (38.0, 74.0)	66.00 (54.0, 78.0)
Age group, *n* (%)			
< 65 years		3 (27.3%)	3 (50.0%)
≥ 65 years		8 (72.7%)	3 (50.0%)
Sex, *n* (%)			
Male		5 (45.5%)	6 (100%)
Female		6 (54.5%)	0 (0%)
Myelofibrosis disease type, *n* (%)			
Primary myelofibrosis		6 (54.5%)	3 (50.0%)
Post-PV myelofibrosis		3 (27.3%)	0 (0%)
Post-ET myelofibrosis		2 (18.2%)	3 (50.0%)
Prior JAK inhibitor therapy			
Median duration, weeks (range)		96.86 (25.0, 400.6)	30.00 (12.1, 165.9)
Ongoing JAK inhibitor at screening, *n* (%)		3 (27.3%)	1 (16.7%)
DIPSS prognostic risk category, *n* (%)			
Intermediate-1		2 (18.2%)	0 (0%)
Intermediate-2		7 (63.6%)	6 (100%)
High		2 (18.2%)	0 (0%)
ECOG performance status, *n* (%)			
0		4 (36.4%)	2 (33.3%)
1		7 (63.6%)	4 (66.7%)
2		0 (0%)	0 (0%)
TSS at baseline, mean (SD)^a^		25. 8 (12.98)	27.4 (14.31)
Central lab spleen volume (cm^3^), mean (SD)		1966.9 (1047.92)	1247.3 (649.76)
Transfusion dependence			
Transfusion independent^b^, *n* (%)		1 (9.1%)	0 (0%)
Transfusion dependent^c^, *n* (%)		2 (18.2%)	3 (50.0%)
Transfusion requiring^d^, *n* (%)		8 (72.7%)	3 (50.0%)
RBC units transfused ≤ 8 weeks before randomized treatment, n (%)^e^			
0		4 (36.4%)	0 (0%)
1–4		5 (45.5%)	5 (83.3%)
≥ 5		2 (18.2%)	1 (16.7%)
Hemoglobin (g/dL), mean (SD)		7.93 (0.79)	7.52 (0.51)
Platelet count (× 10^9^/L), mean (SD)		149.09 (110.36)	111.67 (94.22)

^a^TSS was assessed using Myelofibrosis Symptom Assessment Form v4.0. ^b^The percentage of patients with TI in the 12 weeks prior to completion of the 24-week randomized-treatment period (84 consecutive days). ^c^Defined as four or more RBC units transfused in the 8 weeks prior to the first dose of study drug. ^d^Not meeting definition of TI or TD. ^e^Data were from the case report form. DAN, danazol; DIPSS, Dynamic International Prognostic Scoring System; ECOG, Eastern Cooperative Oncology Group; JAK, Janus kinase; MMB, momelotinib; post-ET, post-essential thrombocythemia; post-PV, post-polycythemia vera; RBC, red blood cell; SD, standard deviation; TI, transfusion independent; TSS, total symptom score

### Endpoint outcomes

**Fig. 2:**
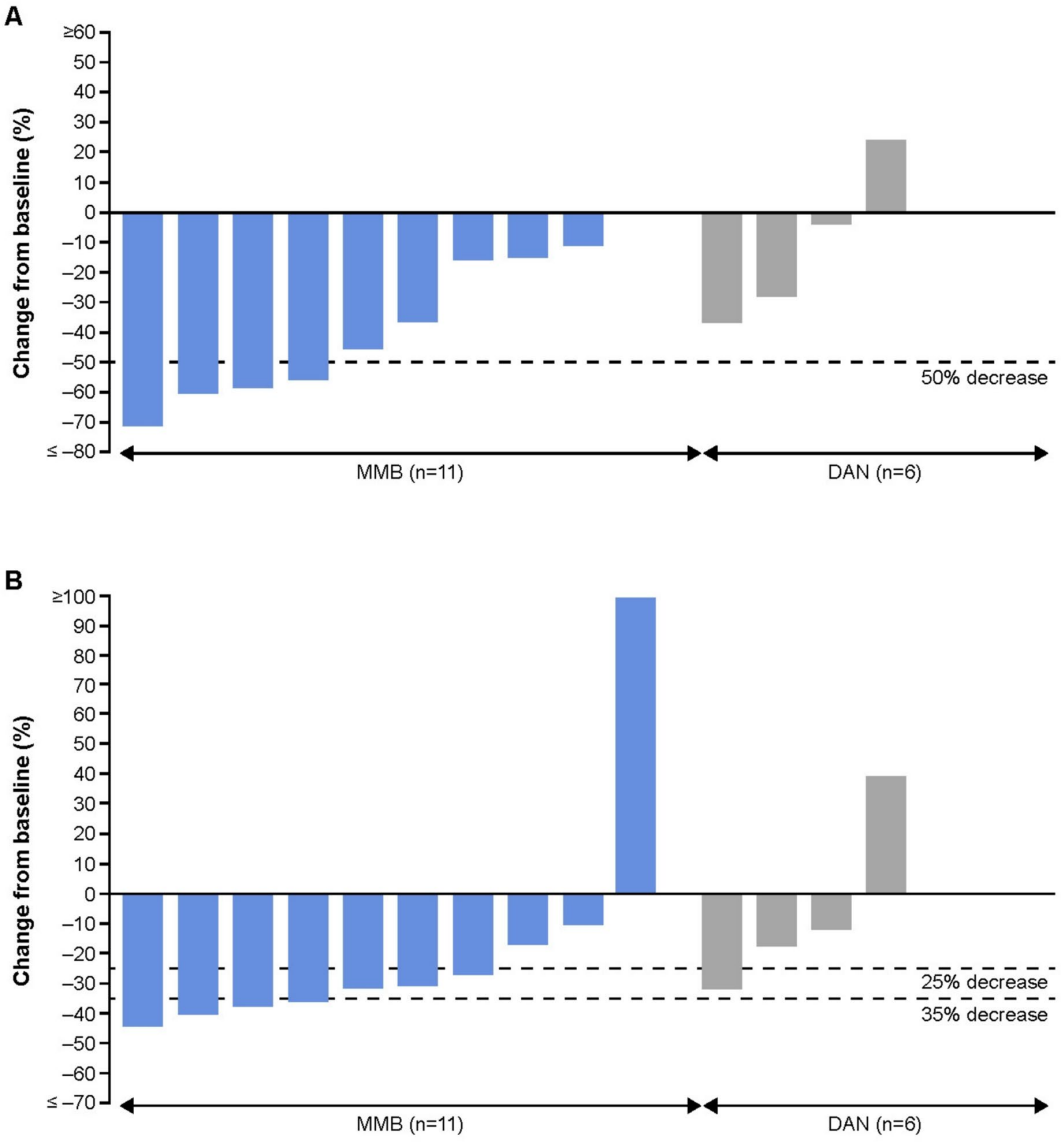
Percent change in (a) total symptom scores and (b) splenic volume at Week 24 post-dose for individual patients DAN, danazol; MMB, momelotinib

**Fig. 3:**
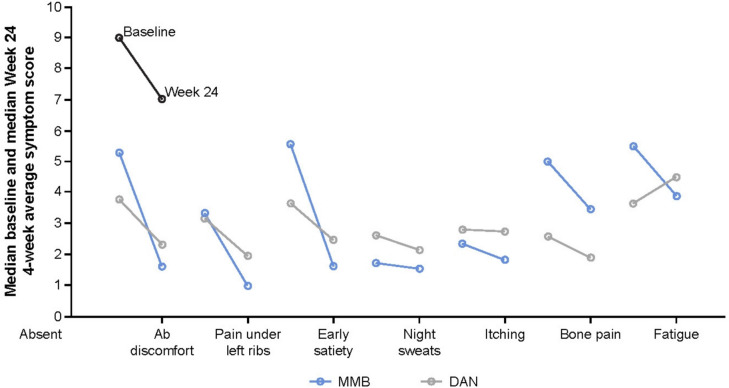
Median MFSAF symptom-scores at baseline and Week 24 Analysis includes patients with both baseline and Week 24 data available. DAN, danazol; MFSAF, Myelofibrosis Symptom Assessment Form; MMB, momelotinib

TSS response rate was 36.4% (4/11) and 0% (0/6) in the momelotinib and danazol groups, respectively (greater proportion difference, 33.3%; 95% CI, −20.0, 86.68) (Table 2, Figs. 2a, 3).

**Table 2:** Efficacy outcomes at Week 24

Efficacy endpoints	MMB *n* = 11	DAN *n* = 6	Difference^a^ (95% CI)
TSS response, *n* (%)	4 (36.4)	0.0	33.3 (− 20.0, 86.7)
TI rate, *n* (%)	7 (63.6)	0.0	100.0^b^ (58.4, 141.6)
SRR (≥ 25% reduction), *n* (%)	7 (63.6)	1 (16.7)	33.3 (− 20.0, 86.7)
Mean TSS change from baseline (SD)	− 11.51 (8.92)	− 3.59 (6.26)	N/A
LS mean TSS change from baseline (SD)	− 9.52 (2.78)	− 9.19 (4.08)	− 0.34^c^ (− 10.9, 10.3)
SRR (≥ 35% reduction), *n* (%)	4 (36.4)	0.0	33.3 (− 20.0, 86.7)
Rate of zero transfusions, *n* (%)	8 (72.7)	0.0	100.0 (100, 100)

^a^Differences in TSS response rate, SRRs and rate of zero transfusions were based on a stratified Cochran–Mantel–Haenszel test; ^b^Non-inferiority proportion difference; ^c^Least squares mean difference. CI, confidence interval; DAN, danazol; LS, least square; MMB, momelotinib; N/A, not available; SD, standard deviation; SRR, splenic response rate; TI, transfusion independence; TSS, total symptom score

At Week 24, the TI rate was 63.6% (7/11; 95% CI, 30.79, 89.07) and 0% (0/6; 95% CI, 0.00, 45.93) in the momelotinib and danazol groups, respectively*.* SRR (≥ 25% reduction) was 63.6% (7/11; 95% CI, 30.79, 89.07) and 16.7% (1/6; 95% CI, 0.42, 64.12) in the momelotinib and danazol group, respectively; SRR (≥ 35% reduction) was 36.4% (4/11; 95% CI, 10.93, 69.21) in the momelotinib group and 0% (0/6; 95% CI, 0.00, 45.93) in the danazol group (Table 2, Fig. 2b).

At Week 24, the least squares (LS) mean (standard error [SE]) change in TSS from baseline was − 9.52 (2.78) and − 9.19 (4.08) in the momelotinib and danazol groups, respectively (difference, − 0.34; 95% CI, − 10.92, 10.25). Although the difference in LS means was small, the decrease in individual item scores was greater in the momelotinib group than in the danazol group (Table 2, Fig. 3).

At Week 24, the proportion of patients who had zero RBC transfusions was 72.7% (8/11; 95% CI, 39.03, 93.98) and 0.0% (0/6; 95% CI, 0.00, 45.93) in the momelotinib and danazol groups, respectively (Table 2). In the momelotinib group, 18.2% (2/11) of patients were TD and 9.1% (1/11) of patients were TI at baseline; 72.7% (8/11) of patients were transfusion requiring (TR [not meeting the definition of TI or TD]). At Week 24, 0.0% (0/11), 63.6% (7/11), and 36.4% (4/11) of patients were TD, TI, and TR, respectively. In the danazol group, 50.0% (3/6), 0.0% (0/6), and 50.0% (3/6) of patients were TD, TI, and TR at baseline, respectively; there was no change in transfusion status at Week 24. In the momelotinib group, none of the patients with TD at baseline (18.2% [2/11]) achieved TI at Week 24, but both became TR at Week 24; of the eight patients who were TR at baseline, six converted to TI and two remained TR. The patient in the momelotinib arm who was TI at baseline remained TI at Week 24 (100% [1/1]; 95% CI 2.50, 100.00).

Ten and three patients continued or crossed over to open-label momelotinib after the randomized-treatment period, with a median follow-up of 49.6 weeks and 37.4 weeks in the momelotinib and danazol groups, respectively. Fatal events were reported in 9.1% (1/11) of patients in the momelotinib group and 16.7% (1/6) patients in the danazol group, both due to leukemic transformation; median OS and LFS were not reached in either group (Supplementary Fig. S1).

### Hemoglobin levels

At Week 4, mean hemoglobin levels increased to 9.1 g/dL from the baseline level of 7.9 g/dL for momelotinib and to 8.7 g/dL from 7.5 g/dL for danazol (Supplementary Fig. S2), with patients treated with momelotinib consistently having higher mean levels of hemoglobin than those treated with danazol. However, after crossing over to open-label momelotinib at Week 24, mean hemoglobin levels in the danazol group increased from 7.7 to 9.5 g/dL after 20 weeks.

### Safety

No new safety signals were identified in this sub-analysis compared with the overall intention-to-treat (ITT) cohort of the MOMENTUM trial (Table 3). During the 24-week randomized period, all patients reported at least one TEAE, most commonly constipation, hyperkalemia, nausea, peripheral edema, and pruritis (17.6% [3/17] each across both groups).

**Table 3:** Summary of TEAEs during the 24-week randomized period

Patients with at least one event, *n* (%)	MMB *n* = 11	DAN *n* = 6
TEAE	11 (100)	6 (100)
Grade ≥ 3 TEAE	4 (36.4)	4 (66.7)
TEAE related to the study treatment	7 (63.6)	2 (33.3)
Grade ≥ 3 TEAE related to the study treatment^a^	2 (18.2)	1 (16.7)
TEAE leading to treatment interruption and/or dose reduction	2 (18.2)	1 (16.7)
TEAE leading to permanent discontinuation of the study treatment	0 (0.0)	2 (33.3)
Serious TEAE	3 (27.3)	3 (50.0)
Serious TEAE related to the study treatment	1 (9.1)	1 (16.7)
Fatal TEAE	1 (9.1)	1 (16.7)
Most common TEAEs, *n* (%)		
Peripheral edema	2 (18.2)	1 (16.7)
Dizziness	2 (18.2)	0 (0)
Diarrhea	2 (18.2)	0 (0)
Fluid overload	2 (18.2)	0 (0)
Hyperuricemia	2 (18.2)	0 (0)
Vomiting	2 (18.2)	0 (0)
Constipation	1 (9.1)	2 (33.3)
Hyperkalemia	1 (9.1)	2 (33.3)
Nausea	1 (9.1)	2 (33.3)
Pruritis	1 (9.1)	2 (33.3)
Increased alanine aminotransferase	0 (0)	2 (33.3)
Increased aspartate aminotransferase	0 (0)	2 (33.3)
Increased creatinine	0 (0)	2 (33.3)

^a^TEAE assessed as related to the study treatment by investigator. DAN, danazol; MMB, momelotinib; TEAE, treatment-emergent adverse event

In the momelotinib group, the most common TEAEs were peripheral edema, diarrhea, dizziness, fluid overload, hyperuricemia, and vomiting (18.2% [2/11] each). In the danazol group, constipation, hyperkalemia, nausea, pruritis, and increased alanine aminotransferase, aspartate aminotransferase, and blood creatinine were the most common TEAEs (33.3% [2/6] each).

Grade ≥ 3 TEAEs were reported in 36.4% (4/11) and 66.7% (4/6) of the momelotinib and danazol groups, respectively, including fluid overload in 18.2% (2/11) of patients in the momelotinib group; one patient in the momelotinib group reported grade ≥ 3 anemia. No patients reported grade ≥ 3 thrombocytopenia or peripheral neuropathy.

TEAEs led to treatment interruption and/or dose reduction in 18.2% (2/11) and 16.7% (1/6) of patients in the momelotinib and danazol groups, respectively. TEAEs led to discontinuation of the study treatment in two patients in the danazol group: one experienced increased alanine aminotransferase and the other experienced increased aspartate aminotransferase.

## Discussion

Consistent with the ITT population of the MOMENTUM trial [20], this sub-analysis showed that, compared with danazol, momelotinib improved splenomegaly, symptoms, and anemia associated with primary MF, post-PV/ET MF in Asian patients previously treated with JAK inhibitors. Notably, efficacy at Week 24 in the momelotinib group was numerically greater in this Asian subpopulation than in the overall population, including TSS response rate (36.4% and 25%, respectively), reduction in splenic volume (≥ 25% reduction: 63.6% and 39%; ≥ 35% reduction: 36.4% and 22%), change in TSS (LS mean − 9.52 and − 9.36), and TI rate (63.6% and 30%)[20]. As this is a post hoc analysis of the MOMENTUM trial where the number of Asian patients was small, the sample size should be considered when interpreting these data.

The primary endpoint of TSS response rate was higher in the momelotinib group than the danazol group in this sub-analysis (36.4% [4/11] vs. 0% [0/6]). This trend was also observed in the primary MOMENTUM analysis where patients in the momelotinib group had a higher TSS response rate than those in the danazol group (24.6% [32/130] vs. 9.2% [6/65]) [20].

For the secondary endpoints, the results of this sub-analysis also aligned with that of the primary MOMENTUM analysis. SRR (≥ 35% reduction) was greater in the momelotinib group than the danazol group (36.4% [4/11] vs. 0% [0/6]), aligning with the primary analysis (22.3% [29/130] vs. 3.1% [2/65]) [20].

In this sub-analysis, a greater LS mean (SE) change in TSS at Week 24 from baseline was observed in the momelotinib group than in the danazol group (− 9.5 [2.78] vs. − 9.2 [4.08]), similar to the primary analyses (LS mean change: − 11.5 for momelotinib vs. − 3.9 for danazol; LS mean difference: − 6.2 [95% CI: − 10.0, − 2.4, *p* = 0.0014]) [20].

The TI rate at Week 24 in this sub-analysis was higher in the momelotinib group than the danazol group (63.6% [7/11] vs. 0% [0/6]), aligning with the results of the overall cohort (30.0% [39/130] for momelotinib vs. 20.0% [13/65] for danazol) [20]. In this analysis, there were fewer patients with TD at Week 24 in the momelotinib group (0% [0/11]) than the danazol group (50% [3/6]). Together, these findings demonstrate the potential efficacy of momelotinib in treating anemia and reducing the transfusion burden on patients with MF. TD is associated with lower functioning and health-related QoL; reducing TD in patients with MF can improve QoL and prognoses compared with patients who remain transfusion dependent [23, 24]. For patients with MF living in Asia, particularly Southeast Asia and China, reducing TD is important due to the limited blood supply and access to health care [25–27].

In both this sub-analysis and the primary analysis, fewer deaths occurred in the momelotinib group than in the danazol group (9.1% [1/11] vs. 16.7% [1/6] and 19.2% [25/130] vs. 24.6% [16/65], [20], respectively); median OS was not reached in either group in this analysis.

There was one LFS event in each group, after a median follow-up of 49.6 weeks and 37.4 weeks in the momelotinib and danazol groups, respectively; each event was fatal in this sub-analysis. In the primary analysis, leukemic transformation events occurred in 2% (3/130) and 6% (4/65) in the momelotinib and danazol group, respectively; median LFS was not reached in either group [20].

The safety profile was consistent with the overall ITT population [20], with no unusual or unexpected AEs in this patient population. AEs were primarily gastrointestinal and hematologic, and manageable; few patients required dose reductions for safety. Although one patient reported grade ≥ 3 anemia in the momelotinib group, the risk of cytopenia was low in the momelotinib and danazol groups despite the high mean relative dose intensities for both groups (96.2% vs. 94.1%); however, one patient (9.1%) treated with momelotinib required dose adjustments due to thrombocytopenia. No patients reported peripheral neuropathy.

In addition to approvals in the United States [28], Europe [29], and the United Kingdom [30], momelotinib has recently been approved by the Ministry of Health, Labour and Welfare, Japan, for patients with MF and anemia [31] based on the pivotal phase 3 trials SIMPLIFY-1 [21] and MOMENTUM [20]. Many therapies, including anticancer therapies, have demonstrated racial and ethnic differences in response and safety [32, 33]. In particular, Asian patients have pharmacogenetic variations that may predispose them to reduced clinical benefits and increased risk of toxicity with some anticancer therapies, highlighting the importance of analyzing the efficacy and safety of new therapies in this population [32, 33]. Together with the Japanese subgroup analysis of the SIMPLIFY-1 trial [34], this analysis of the Asian subpopulation of the MOMENTUM study demonstrated that the efficacy and safety of momelotinib aligned with the overall ITT population [20], supporting its use in Asian patients.

## Limitations

The primary limitation of this sub-analysis is its small sample size, so findings must be confirmed by future investigations. Results may not be geographically representative of the Asian continent as the study population only comprised a limited number of countries. As a sub-analysis of the MOMENTUM trial, this study inherits any limitations in the original study design. Notably, a lack of long-term comparison of survival between treatment groups due to the crossover study design, and the use of danazol as the comparator drug, which may be a limitation as it is typically only used to manage anemia in MF, and not splenomegaly and associated symptoms; however, momelotinib still outperformed danazol in the treatment of MF-associated anemia. Patients and investigators may have also tried to predict their treatment assignment based on previous JAK-inhibitor experience. Finally, as a post hoc analysis of a larger study, this was designed to be descriptive, with no formal hypothesis testing; results from this sub-analysis should be validated in future studies.

## Conclusion

Among the JAK inhibitor-experienced Asian subpopulation with symptomatic and anemic MF from the MOMENTUM trial, momelotinib was associated with clinically significant improvements in MF-associated symptoms, anemia measures, and spleen size, with favorable safety compared with danazol, which were generally consistent with the overall ITT population. These data support momelotinib as a potentially effective treatment option for Asian patients with symptomatic and anemic MF.

## Supplementary Information

Below is the link to the electronic supplementary material.

Supplementary file1 (DOCX 284 kb)

## Data Availability

For requests for access to anonymized subject level data, please contact corresponding author.
