# Supplementary material for: Efficacy and safety of momelotinib in Janus kinase inhibitor-experienced Asian patients with myelofibrosis and anemia
Source: Int J Hematol. 2025 Jul 21;122(5):660–70. doi: 10.1007/s12185-025-04037-6 (PMC12572043; doi:10.1007/s12185-025-04037-6)
Supplement: Supplementary file 1 — Supplementary file1 (DOCX 284 kb) [file 12185_2025_4037_MOESM1_ESM.docx]

**ONLINE RESOURCES**

**Title**

Efficacy and safety of momelotinib versus danazol in JAK inhibitor-experienced patients with myelofibrosis and anemia: Asian subgroup analysis of the MOMENTUM trial

**Supplementary Figure 1. Kaplan–Meier curves for overall survival by treatment group**


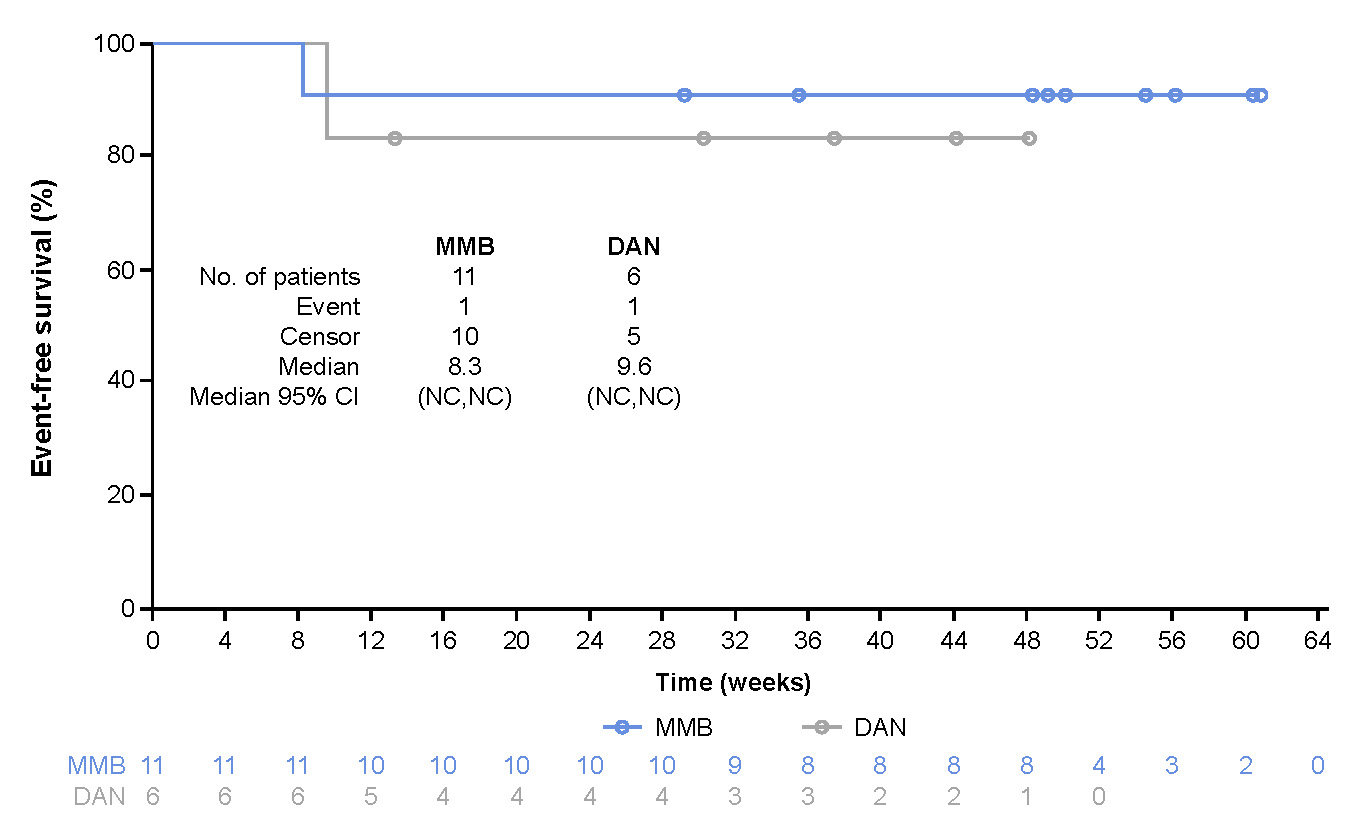


Ο = censored observation. DAN, danazol; MMB, momelotinib; NC, not computable

**Supplementary Figure 2. Mean hemoglobin levels over time by treatment group**

**
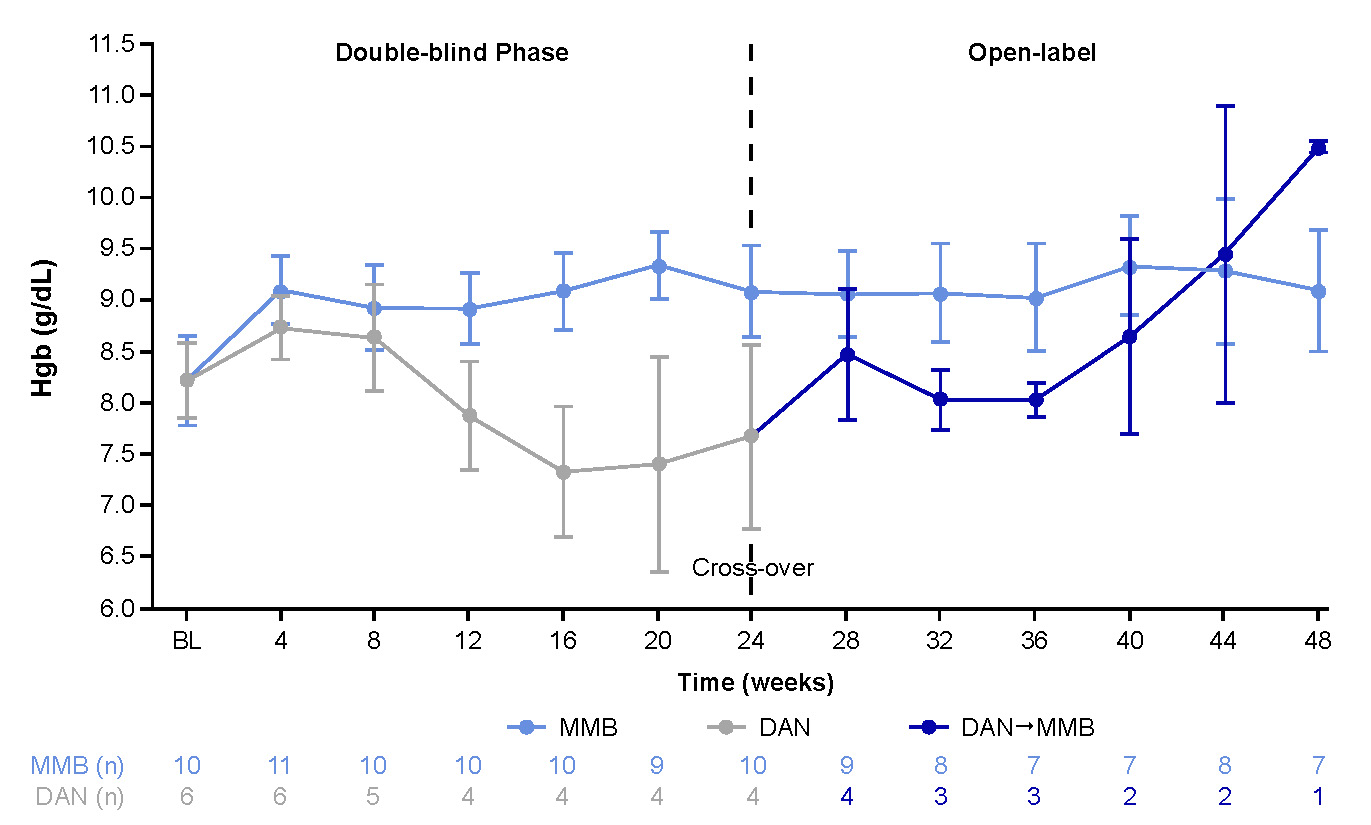
**DAN, danazol; Hgb, hemoglobin; MMB, momelotinib
